# Estimating global ocean heat content from tidal magnetic satellite observations

**DOI:** 10.1038/s41598-019-44397-8

**Published:** 2019-05-27

**Authors:** Christopher Irrgang, Jan Saynisch, Maik Thomas

**Affiliations:** 1 Helmholtz Centre Potsdam, GFZ German Research Centre for Geosciences, Section 1.3, Earth System Modelling, Telegrafenberg A20, 14473 Potsdam, Germany; 2 Institute of Meteorology, Freie Universität Berlin, Carl-Heinrich-Becker-Weg 6-10, 12165 Berlin, Germany

**Keywords:** Computational science, Physical oceanography

## Abstract

Ocean tides generate electromagnetic (EM) signals that are emitted into space and can be recorded with low-Earth-orbiting satellites. Observations of oceanic EM signals contain aggregated information about global transports of water, heat, and salinity. We utilize an artificial neural network (ANN) as a non-linear inversion scheme and demonstrate how to infer ocean heat content (OHC) estimates from magnetic signals of the lunar semi-diurnal (M2) tide. The ANN is trained using monthly OHC estimates based on oceanographic *in*-*situ* data from 1990–2015 and the corresponding computed tidal magnetic fields at satellite altitude. We show that the ANN can closely recover inter-annual and decadal OHC variations from simulated tidal magnetic signals. Using the trained ANN, we present the first OHC estimates from recently extracted tidal magnetic satellite observations. Such space-borne OHC estimates can complement the already existing *in*-*situ* measurements of upper ocean temperature and can also allow insights into abyssal OHC, where *in*-*situ* data are still very scarce.

## Introduction

The world ocean absorbs and stores huge amounts of heat due to the present Earth’s energy imbalance (EEI) and the ongoing global warming^1^. Since more than 90% of the EEI is stored in the inert ocean^2–4^, estimating the ocean heat content (OHC) has become a crucial task for monitoring and understanding the Earth’s changing climate from inter-annual to multi-decadal time scales. Both, *in*-*situ* measurements and ocean reanalyses agree that the global ocean heat content is steadily increasing^5–7^ and was continuously involving deeper regions of the ocean during the last three decades^8,9^. The consequences for the Earth’s climate are manifold. One prominent example is the associated thermosteric sea-level rise, which accounts for approximately one third of the observed global mean sea-level rise^10,11^.

**Figure 1:**
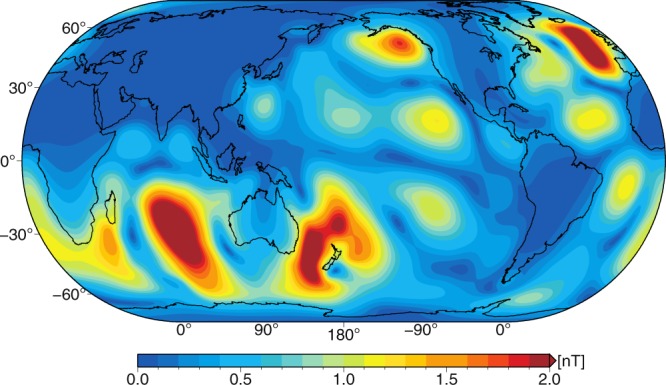
Absolute radial component of the periodic M2 tidal magnetic field at a satellite altitude of 430 km above sea surface.

Ocean temperature and salinity are the major variables that determine the electrical conductivity of sea-water. In the presence of the geomagnetic core field, the moving and electrically conducting sea-water generates electric currents that, in turn, induce weak magnetic signals in and outside of the ocean^12,13^. Especially the magnetic field generated by the lunar semi-diurnal ocean tide (M2, see Fig. 1) has gained attention, since its periodic signals were detected in land observatories^14,15^, in ocean bottom measurements^16^, and also by low-Earth-orbiting satellites^17–19^. Space-borne observations of oceanic magnetic signals are of high value for oceanographic applications, as they contain nearly global information on combined transports of water, heat, and salinity in the ocean. Tidal magnetic signals, in particular, are generated with non-changing (and precisely known) periodicity during the time scales of interest. Thus, superimposed trends and variations of these magnetic signals are largely attributable to changes in sea-water conductivity, which depends on oceanic heat and salinity distribution (see also Ohm’s Law in the methods section). In this context, numerical forward simulations by Saynisch *et al*. have shown that temporal anomalies in the otherwise periodic M2 tidal magnetic field can be linked to climate change processes like ocean warming^20,21^. Recently, the non-linear relation between the global OHC and the ocean’s electrical conductivity was examined and the high correlation between the two variables was emphasized^22^.

**Figure 2:**
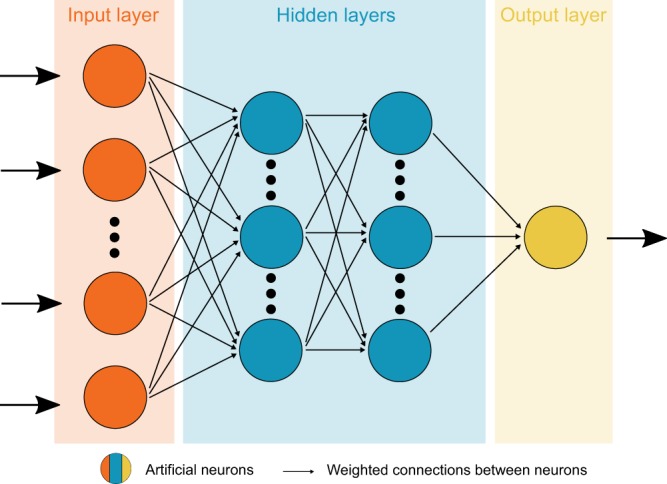
Sketch of a feed-forward artificial neural network (ANN) with one input layer, two hidden layers, and one output layer. Training (or validation) data are successively passed through all neurons of the ANN from the input layer to the output layer. The ANN is trained to estimate the global ocean heat content (output layer) based on the corresponding M2 tidal magnetic field (input layer).

In this study, we show that estimates of the global OHC can be inferred from the space-borne M2 tidal magnetic field with an artificial neural network (ANN, see Fig. 2). ANNs build one branch of machine learning techniques and were proposed as a powerful tool for analyzing and predicting multivariate and non-linear relationships in oceanography^23,24^ and remote sensing^25^. We setup and use a feed-forward ANN as a non-linear inversion scheme to recover and predict the increasing global OHC from the corresponding temporal anomalies in the M2 tidal magnetic field. To train an ANN for this task, we build an experiment environment that combines numerical simulations with real-world observations. In particular, M2 tidal magnetic fields at satellite altitude are derived with an electromagnetic (EM) induction solver^26^ from a global tide model^27^ and an ensemble of four different data products of monthly varying upper ocean (0–2000 m) temperature and salinity during the 1990–2015 time period. The temperature and salinity data products (denoted CORA5^28^, JMA^29^, EN4^30^, and IAP^9^; see details in the Materials and Methods section) are compiled by different centres and include *in*-*situ* measurements from Argo floats, CTD (conductivity, temperature depth) instruments, XBT (expendable bathythermograph), MBT (mechanical bathythermograph), gliders, and others. The respective estimates of monthly global OHC are derived from the same temperature data. In combination, these data pairs are used to train the ANN, i.e., M2 tidal magnetic fields as inputs and corresponding OHC as outputs. This training routine allows the ANN to learn the non-linear relationship between the tidal magnetic signals and global OHC. Ultimately, the trained ANN is applied to derive OHC estimates from recently extracted global satellite observations of the M2 tidal magnetic field.

## Results and Discussion

**Figure 3:**
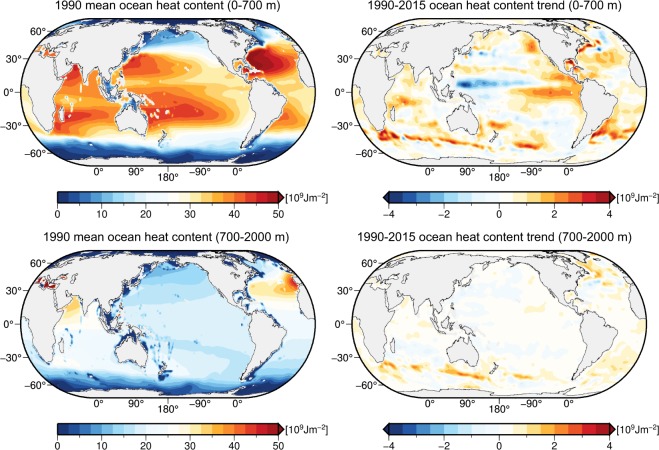
Ensemble (CORA5, JMA, EN4, IAP) mean areal distributions of the upper ocean heat content for the 0–700 m and 700–2000 m ocean layers in 1990 (left column) and corresponding linear ocean heat content trends for the 1990–2015 time period (right column).

**Figure 4:**
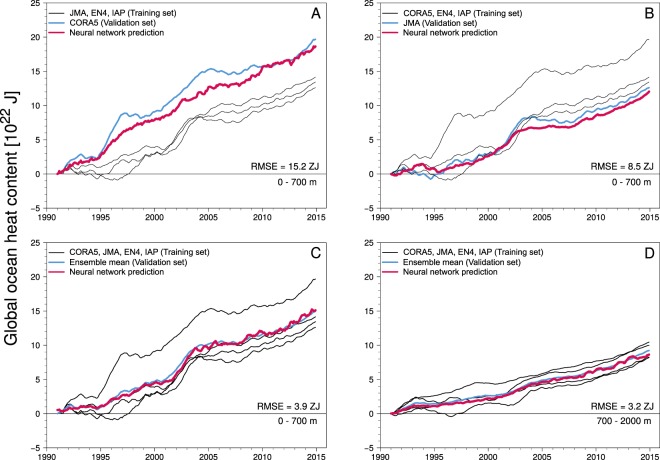
Global ocean heat content (OHC) predictions based on simulated M2 tidal magnetic fields. OHC values are shown w.r.t. the 1990 mean OHC. Panels (A and B) show the recovery of highest and lowest OHC trajectory in the 0–700 m ocean layer after training the ANN with the respective remaining three data products. Panels (C and D) show the recovery of the ensemble mean OHC in the 0–700 m and 700–2000 m ocean layers after training the ANN with all four data products. RMS errors (1 ZJ = 10^21^ J) are given for the offset between the ANN prediction and the validation set.

Areal maps of the estimated annual ensemble mean ocean heat content (OHC) in 1990 and of the respective OHC trends for 1990–2015 are depicted in Fig. 3 for the upper 0–700 m and 700–2000 m ocean layers. The corresponding global OHC trajectories w.r.t. the 1990 mean are shown as black curves in Fig. 4. The apparent ocean warming is visible in almost all regions of the world ocean and is subject to extensive analyses (see, e.g., the coverage of Rhein *et al*.^3^), which are not part of this study. Here, we emphasize that the increasing OHC (Figs 3 and 4) is encoded as variations of the periodic M2 tidal magnetic field that, consequently, can be a valuable observation operator. A detailed discussion of the expected magnetic field anomalies due to ocean warming was already conducted by Saynisch *et al*.^21^.

The M2 tidal magnetic fields as predicted by numerical simulations and as recovered from (space-borne) observations have reached good agreement^18,26,31^. Recently, Sabaka *et al*. extracted the M2 tidal magnetic field from around 20 months of high-resolution satellite observations^18^, which were recorded by the Swarm satellite trio of the European Space Agency (ESA)^32^. Since the corresponding OHC is contained in such space-borne global fields in a temporally averaged sense, we do not aim to relate the highly variable monthly variations of the OHC anomalies to the M2 magnetic field. Instead, the estimated global OHC anomalies are smoothed with a centered 24-month running mean window to only include inter-annual temporal variations and decadal trends (curves in Fig. 4), and to maximize the consistency between the numerically simulated and the satellite-based observations of the tidal magnetic signals.

Six experiments are performed, in which the ANN is trained to recover the increasing OHC due to ocean warming in the 0–700 m and in the 700–2000 m ocean layers from the M2 magnetic field (see Table 1). The details of the ANN setup and training are described in the Materials and Methods section.

**Table 1:** Experiment setups for training and testing the artificial neural network.

Experiment	Ocean layer [m]	Training set	Validation set	RMS error [ZJ]
A	0–700	JMA, EN4, IAP	CORA5	15.2
B	0–700	CORA5, EN4, IAP	JMA	8.5
C	0–700	CORA5, JMA, EN4, IAP	Ensemble mean	3.9
D	700–2000	CORA5, JMA, EN4, IAP	Ensemble mean	3.2
E	0–700	CORA5, JMA, EN4, IAP	CM5, CI	—
F	700–2000	CORA5, JMA, EN4, IAP	CM5, CI	—

**Figure 5:**
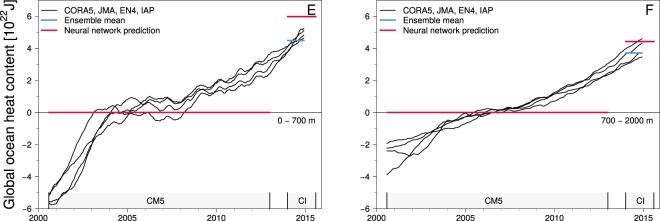
Global ocean heat content (OHC) predictions based on satellite measurements of the M2 tidal magnetic field from the CM5 and CI products. Panel (E) shows the predictions for the 0–700 m and panel (F) for the 700–2000 m ocean layer. The gray boxes indicate the temporal data coverage of the respective satellite measurements. All OHC values are shown w.r.t. the mean values over the CM5 time period. Note that the ensemble mean and the neural network prediction overlap for the CM5 time period.

Results are shown in Fig. 4 for experiments A–D and in Fig. 5 for experiments E and F. RMS errors (1 ZJ = 10^21^ J) are reported for the offset between the neural network prediction and the validation set.

The first two experiments (panels A and B in Fig. 4) serve as extreme tests to examine the ANN’s ability to generalize its prediction skill beyond the known training data. For this purpose, the ANN is trained with three out of the four OHC products and the corresponding M2 tidal magnetic fields. The respective omitted fourth product, i.e., the overall highest and the overall lowest OHC (blue curves in panels A and B in Fig. 4), is used for the validation. Note that in all experiments the ANN prediction is solely derived from tidal magnetic signals without any knowledge of the underlying temperature distribution in the ocean, or time points in the 1990–2015 period. The root-mean-square (rms) errors between the ANN prediction and the validation samples amount to 15.2 ZJ (1 ZJ = 10^21^ J) (A) and 8.5 ZJ (B), respectively. Compared to the estimated maximum increase of 196 ZJ during the 1990–2015 period, the ANN is able to recover the long-term OHC trend in the 0–700 m ocean layer in both experiments.

For the second pair of experiments (panels C and D in Fig. 4), we setup a more realistic scenario, in which the unknown (and validation) OHC can be described as a combination of the different training data. The ANN is trained with all four OHC products and respective M2 tidal magnetic fields. The ANN is then applied to recover the products’ ensemble mean OHC in the 0–700 m and 700–2000 m ocean layers (blue curves in panels C and D in Fig. 4), which is a commonly chosen best-guess of the true OHC (see, e.g., Cheng *et al*.^6^). In both experiments, the ANN’s prediction skill is enhanced significantly compared to experiments A and B with rms errors of 3.9 ZJ for the 0–700 m and 3.2 ZJ for the 700–2000 m ocean layers. The maximal offsets between the ANN prediction and the validation data lie within a ±9.1 ZJ (C) and ±7.4 ZJ (D) range, respectively. This improvement results from the increased amount of training data that, in addition, moves the validation set into the knowledge horizon of the ANN. As a consequence, the ANN closely recovers the non-linear inter-annual and decadal mean OHC trajectories with high correlation and explained variance ($\geqslant$0.97). As the performance of the ANN, among other factors, heavily depends on the amount and quality of training data, the reported errors will likely decrease further along with the future extension of measurement trajectories. More importantly, a data series extension can not only result in an improvement of the most recent OHC estimates, but in a better recovery during the entire considered time period. The accurate estimation of the global OHC is still a difficult task that depends on spatio-temporal data distribution, *in*-*situ* measurement errors, and processing techniques. Recently, Boyer *et al*.^33^ reported that OHC uncertainties can amount to more than 20 ZJ and can exceed the inter-annual variability of OHC anomalies. In this context, we can conclude, that the OHC recovery from tidal magnetic signals fits well within the general uncertainty budget of the OHC estimation.

In the final experiments (Fig. 5), the trained and validated ANN from the previous experiments C and D is used to derive OHC estimates from real-world satellite observations of the M2 magnetic field. Two different M2 tidal magnetic fields products (denoted CM5 and CI as by Sabaka *et al*.^18,^^31^) are used, which were derived from satellite observations during two consecutive time periods, i.e., August 2000 to January 2013 (CM5), and 28 November 2013 to 15 August 2015 (CI). The ANN predictions based on the satellite observations generally follow the *in*-*situ* based OHC estimates (Fig. 5). In the 0–700 m layer, the OHC increase between the CI and CM5 time periods amounts to 60.0 ZJ, which is 15.0 ZJ higher compared to the averaged *in*-*situ* based OHC increase (see red and blue horizontal bars in panel E of Fig. 5). For the 700–2000 m layer, the ANN prediction is 7.1 ZJ higher than the *in*-*situ* based estimates in the CI time period. However, the utilized *in*-*situ* based OHC data coverage does not extend over the entire data coverage period of the CI product, which could set the averaged values (blue horizontal bars) to a higher level and, thus, decrease the difference to the ANN prediction. The ongoing efforts to extract tidal magnetic signals from satellite observations with minimal error budgets (see also Sabaka *et al*.^19^) over different time periods could allow further extending the estimation of the global ocean heat content from space.

Space-borne tidal magnetic signals could complement existing *in*-*situ* based measurements for inferring global OHC estimates in several ways. The nearly global coverage of tidal magnetic satellite observations could be utilized for improving estimates of ocean heat in regions where *in*-*situ* data are still very scarce. This is especially interesting for regions covered by ice^4^ since oceanic magnetic signals are emitted through the ice layer. This leads to the possibility of not only estimating the global OHC from tidal magnetic signals, but also to estimating lateral variations of upper OHC as shown in Fig. 3. To enhance the performance of the ANN in this regard, robust magnetic signals from further separable tidal constituents, e.g. N2^18^, could be added to the ANN training. Additionally, auxiliary data, e.g., estimates of satellite measurement errors and noise, the secular variation of the geomagnetic core field, or other EM constituents, could be added to the ANN training to further increase its performance. Another application arises due to the predominantly barotropic source of tidal magnetic signals that, consequently, contain information about oceanic heat from the entire water column. This is promising, since the majority of *in*-*situ* measurements only cover the upper 2000 m of the ocean and, so far, leave abyssal OHC unobserved. This was also identified as a major source of uncertainty of the deep OHC estimation^34^. The need to extend ocean temperature observations to the deep ocean were repeatedly emphasized^5,11,35^, which recently resulted in the deployment of the first Deep Argo floats, which allow measuring ocean temperature down to a depth of 6000 m^36–38^. In this context, the combination of tidal magnetic signals and machine learning could help to overcome the present lack of abyssal *in*-*situ* temperature data. In particular, training an ANN based on the novel Deep Argo measurements could allow utilizing the global tidal magnetic signals to extend deep OHC estimates into regions, where *in*-*situ* data are not yet available.

## Materials and Methods

### Ocean heat content and conductance

Ocean temperature and salinity records are used in the form of monthly-averaged global grids for the time period of 1990 to 2015 from four data sources: Coriolis Ocean database for ReAnalysis (CORA5^28^), Japan Meteorological Agency (JMA version 7.2^29^), Met Office Hadley Centre for Climate Change (EN4.2.1^30^), and Institute of Atmospheric Physics (IAP CZ16v3^9^). The data products include *in*-*situ* measurements from various sources, e.g., Argo floats, CTD, XBT, MBT, gliders, and others, which in combination extent from the sea surface to a depth of 2000 m^39^. For this data product ensemble, monthly 1° × 1° areal maps of global upper ocean heat content are derived from the temperature data for the 0–700 m and the 700–2000 m depths (Fig. 3). Additionally, the resulting global ocean heat content w.r.t. to the 1990 mean values are calculated (Fig. 4) and smoothed with a 24-month running mean window to remove high-frequency seasonal variability and to maximize the consistency between simulated and measured tidal magnetic signals. The sea-water conductivity *σ* = *σ*(*T*, *S*) in the upper 2000 m is calculated from the monthly varying temperature (T) and salinity (S) fields following^40^. In the deep ocean below 2000 m, the sea-water conductivity is derived from the Ocean Model for Circulation and Tides^41^ as described by Irrgang *et al*.^42^. In combination, the ocean conductance is used to account for the present upper ocean heating (right panel of Fig. 3) in the tidal electromagnetic induction process.

### Tidal electromagnetic induction

Global 1° × 1° M2 tidal magnetic fields are calculated at a satellite altitude of 430 km above the sea surface with the three-dimensional EM induction solver X3DG of Kuvshinov^26^. In particular, we focus on the radial component of the M2 magnetic field (Fig. 1) that is emitted outside of the ocean and observable from space with the Swarm satellite trio from the European Space Agency^18,32^. X3DG solves Maxwell’s equations in the frequency domain with a volume integral equation technique^43^. For this, the solver is provided with input data for the Earth’s electrical conductivity structure and for the tidal electric currents. Below the ocean layer, a global and laterally varying sediment conductance is included by combining sediment thicknesses^44^ with estimates for the corresponding sediment conductivity^45^. A radially symmetric mantle conductivity is included in the form of a vertical profile that follows the results of Püthe *et al*.^46^. The M2 tidal electric currents $\overrightarrow{j}$ are calculated according to Ohm’s law, i.e.,

$${\overrightarrow{j}}_{m,p}={\sigma }_{m,p}\,(\overrightarrow{u}\times \overrightarrow{B}),$$

where *σ*_*m*,*p*_ is the mean sea-water conductivity of product $p\in \{{\rm{CORA}}5,{\rm{JMA}},{\rm{EN}}4,{\rm{IAP}}\}$ in month *m* during 1990 and 2015, $\overrightarrow{u}$ is the M2 tidal transport based on HAMTIDE12^27^, and $\overrightarrow{B}$ is the geomagnetic core field based on the International Geomagnetic Reference Field (IGRF-12)^47^. Given the monthly varying sea-water conductivity *σ*_*m*,*p*_ derived from the different data products, 1152 global fields (288 monthly fields for each of the four products) of the M2 tidal magnetic field at satellite altitude are calculated for the period of 1990 to 2015. Consequently, the upper ocean warming as shown in Figs 3 and 4 is encoded in the temporal changes of the otherwise periodic M2 tidal magnetic signals. Besides the numerically calculated M2 magnetic fields we incorporate two global fields with space-borne observations of M2 magnetic signals. These were extracted from two consecutive time periods, i.e., August 2000 to January 2013 [31, denoted CM5], and 28 November 2013 to 15 August 2015 [18, denoted CI].

### Artificial neural network

The machine learning technique is based on a feed-forward artificial neural network (see sketch in Fig. 2), hereafter called ANN. The ANN is a multilayer perceptron consisting of connected processing nodes (neurons) that are arranged in an input layer, hidden layers, and an output layer^48^. Input data are passed through the ANN and processed by the neurons according to

$${y}_{j}=\phi (\sum _{i=1}^{n}\,{w}_{ji}{x}_{i}+{b}_{j}),$$

where $x\in {{\mathbb{R}}}^{n}$ is the (normalized) input vector with length *n* of the neuron, ${y}_{j}\in {\mathbb{R}}$ is the output of the *j*-th neuron, ${w}_{j}\in {{\mathbb{R}}}^{n}$ are the connection weights of the respective input streams of the *j*-th neuron, ${b}_{j}\in {\mathbb{R}}$ is the activation (threshold) parameter, and *φ*(·) is the (usually) non-linear activation function. In this study, we utilize the H2O deep learning architecture to set up the ANN^49^. We use a network topology with four hidden layers that contain 50, 50, 25, and 10 neurons, respectively, and the Maxout activation function^50^. The ANN is trained to estimate the global ocean heat content from the M2 tidal magnetic field in a supervised learning routine. For this, the data are separated into training and validation sets based on the different ocean temperature products described above (see Table 1). Additionally, only 50% of the wet grid points of the 1° × 1° M2 tidal magnetic fields are considered for the learning process, which results in 19281 input neurons. This is done to keep the computational demand of the learning process feasible. While iteratively exposing a training set to the ANN, the network is learning the non-linear relationship between global ocean heat content and the corresponding M2 magnetic field by adjusting the weights *w*_*ji*_ of the neuronal connections with the widely used back-propagation algorithm^51,52^. After the training, the weights are fixed and new, i.e., unknown, M2 magnetic fields from the validation set can be passed through the ANN. We examine the performance of the ANN by comparing the network’s prediction of the global ocean heat content with the global ocean heat content from the respective validation set (A–D in Table 1 and Fig. 4). In addition to the experiments based on simulated M2 magnetic signals, the trained ANN is used to estimate the global ocean heat content from actual satellite measurements of M2 magnetic signals, which were recovered from two recent consecutive time periods (E–F in Table 1 and Fig. 5).

## Data Availability

The data and products used for this study can be directly obtained from the working groups that provide HAMTIDE12 (https://icdc.cen.uni-hamburg.de/daten/ocean/hamtide.html), IGRF-12 (https://www.ngdc.noaa.gov/IAGA/vmod/igrf.html), CORA5 (via http://www.argo.ucsd.edu/Gridded_fields.html), IAP (ftp://ds1.iap.ac.cn/ftp/cheng/CZ16_v3_IAP_Temperature_gridded_1month_netcdf/), EN4 (https://www.metoffice.gov.uk/hadobs/en4/download-en4-2-1.html), JMA (https://climate.mri-jma.go.jp/pub/ocean/ts/), and H2O (https://www.h2o.ai/h2o/), respectively. Researchers interested in using data from the OMCT may contact Maik Thomas (maik.thomas@gfz-potsdam.de).
